# Nicotinamide Riboside: What It Takes to Incorporate It into RNA

**DOI:** 10.3390/molecules29163788

**Published:** 2024-08-10

**Authors:** Felix Wenzek, Alexander Biallas, Sabine Müller

**Affiliations:** Institute of Biochemistry, University of Greifswald, Felix-Hausdorff-Str. 4, 17487 Greifswald, Germany; felix.wenzek@uni-greifswald.de (F.W.); alexander.biallas@stud.uni-greifswald.de (A.B.)

**Keywords:** co-factor, nicotinamide ribonucleotide, redox reaction, ribozyme, RNA

## Abstract

Nicotinamide is an important functional compound and, in the form of nicotinamide adenine dinucleotide (NAD), is used as a co-factor by protein-based enzymes to catalyze redox reactions. In the context of the RNA world hypothesis, it is therefore reasonable to assume that ancestral ribozymes could have used co-factors such as NAD or its simpler analog nicotinamide riboside (NAR) to catalyze redox reactions. The only described example of such an engineered ribozyme uses a nicotinamide moiety bound to the ribozyme through non-covalent interactions. Covalent attachment of NAR to RNA could be advantageous, but the demonstration of such scenarios to date has suffered from the chemical instability of both NAR and its reduced form, NARH, making their use in oligonucleotide synthesis less straightforward. Here, we review the literature describing the chemical properties of the oxidized and reduced species of NAR, their synthesis, and previous attempts to incorporate either species into RNA. We discuss how to overcome the stability problem and succeed in generating RNA structures incorporating NAR.

## 1. Introduction

In the early stages of life, RNA is thought to have been the sole actor responsible for all catalytic activities and the maintenance of genetic information. Hypothetically, a set of catalysts consisting only of RNA supported all the reactions required for the synthesis of single ribonucleotides, polynucleotides, and other biomolecules [1,2]. At some point, regulation of these ribozyme activities was needed to adapt to fluctuations in, for example, metabolite concentrations or external signals [3,4,5]. This would require the binding of external regulators, which could be amino acids, small peptides, or other molecules, for allosteric control of activity [1]. In addition, ribozymes may have required co-factors for function, as is known for many protein enzymes today. Many co-factors that occur in the modern cell are derivatives of ribonucleotides and are, therefore, considered remnants of the RNA world [6]. Interestingly, RNA is able to support the synthesis of the co-factors coenzyme A (CoA), nicotinamide dinucleotide (NAD), and flavine adenine dinucleotide (FAD). However, this occurs only at the final stage of their respective biosynthetic pathways, when the basic structure of the co-factor is already established [7]. It is, therefore, reasonable to postulate that these co-factors originated separately from RNA in early life through prebiotic chemistry and may have supported RNA function. Initially, ribozymes and co-factors may have formed a complex through non-covalent interactions or through a covalent linkage at the 5′ or 3′ terminus of the ribozyme, possibly giving the ribozyme an evolutionary advantage through extended or simply better functional performance [8,9].

A number of laboratory-developed artificial ribozymes have been created that require a co-factor for activity. One such example is a ribozyme that utilizes covalently bound β-nicotinamide mononucleotide for RNA ligation, a co-factor that has been shown to arise under prebiotic conditions [10,11,12,13]. In modern biological systems, nicotinamide remains an important functional compound, and in the form of NAD, is used as a co-factor by protein-based enzymes for catalyzing redox reactions [14]. Furthermore, the co-factors pyrrolochinolinchinone (PQQ), NAD, and FAD have been demonstrated to exhibit redox activity when complexed with a DNA aptamer [15]. Therefore, it is reasonable to hypothesize that primordial ribozymes could have utilized co-factors such as NAD to catalyze redox reactions. Indeed, through in vitro selection, a ribozyme, designated Ribox02, was successfully generated with an activity analogous to that of an alcohol dehydrogenase (ADH) [16,17].

**Figure 1:**
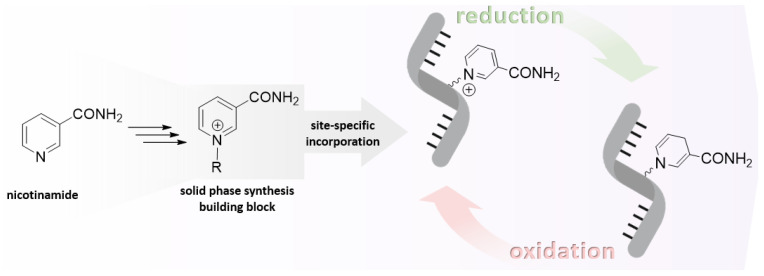
Scheme of the redox cycle of an artificial nicotinamide-containing RNA structure with oxidoreductase activity.

Given their role as a crucial mechanism in prebiotic catalysis, redox reactions supported by ribozymes in conjunction with co-factors such as NAD appear to be a highly plausible avenue of investigation. There are numerous possibilities for the design of redox-active ribozymes. In addition to the non-covalent interaction of the co-factor with the ribozyme, the covalent attachment of the redox co-factor to the ribozyme as a prosthetic group represents a compelling proposition. This could result in the creation of more functional ribozymes that are capable of mimicking their respective enzyme counterparts in modern biochemistry. The gold standard for synthesizing RNA-incorporating modified nucleotides remains the phosphoramidite method. Consequently, it is of great interest to ascertain whether the particular co-factor is compatible with this chemistry. This review will focus on nicotinamide riboside (NAR) as a redox co-factor and address the question of whether it is possible to chemically incorporate it into RNA structures (Figure 1). Attaining this goal would provide further insight into the potential functions of RNA redox catalysis during the early stages of the RNA world. Furthermore, it would facilitate the development of redox ribozymes.

## 2. Stability of Nicotinamide Riboside

The thermal stability of the oxidized and reduced species, namely NAR and NARH, at elevated temperatures rarely presents a challenge during chemical synthesis. Nevertheless, both forms are susceptible to degradation by nucleophiles in either acidic or alkaline environments, whereas they exhibit tolerable stability under neutral or physiological conditions.

### 2.1. Nucleophilic Attack on the Anomeric Carbon and the Pyridinium Moiety Leads to Decomposition of Nicotinamide Riboside under Alkaline Conditions

**Figure 2:**
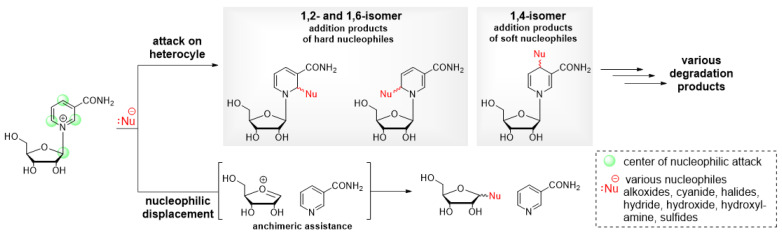
Nucleophilic degradation of NAR by either attack onto the heterocycle or the anomeric carbon.

Nicotinamide riboside (NAR) exhibits remarkable stability in acidic media [18], but due to its intrinsically cationic nature, it is labile under neutral and alkaline conditions by various degradation routes [19], which, for simplicity, have been divided into two major pathways (Figure 2).

**Figure 3:**
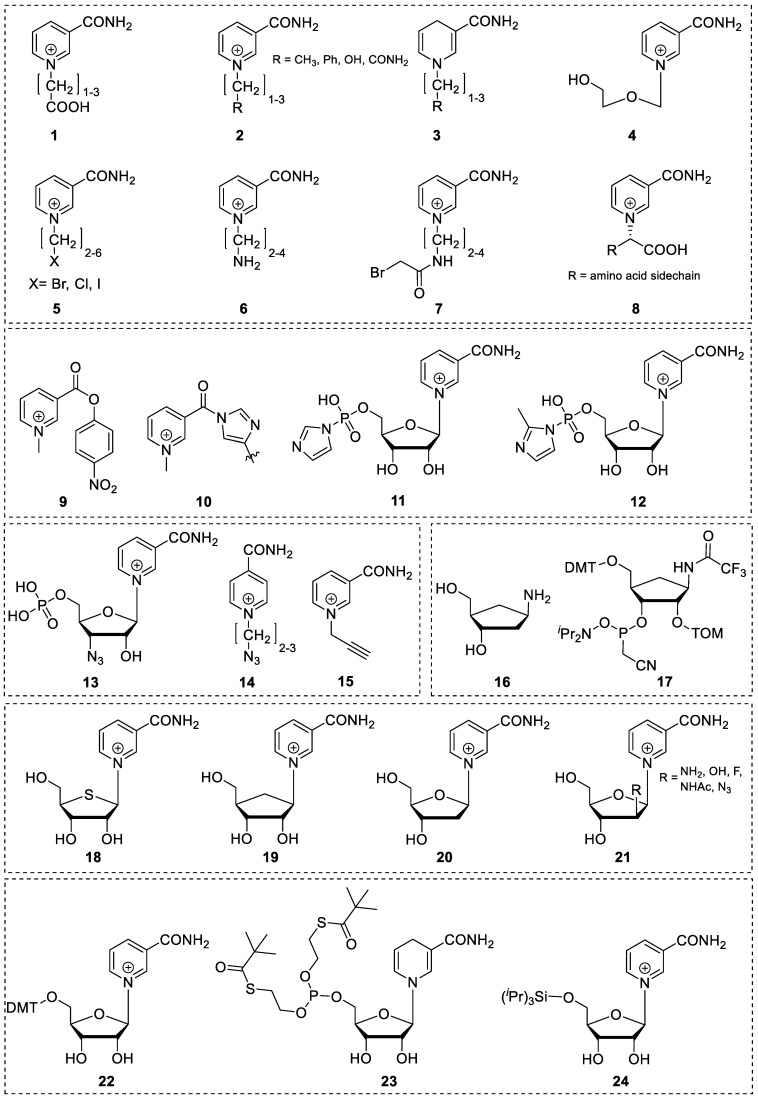
Selection of key nicotinamide-based compounds important for incorporation into RNA structures.

The first pathway entails the cleavage of the glycosidic bond, which occurs because the pyridinium moiety functions as an excellent leaving group in the presence of suitable nucleophiles, such as hydroxide anions. This leads to the formation of nicotinamide and D-ribose. The glycosidic bond cleavage is influenced by temperature, with an increase of approximately 10 °C, resulting in a doubling of the rate. The reaction is facilitated by the assistance of the sugar moiety, which forms an oxocarbenium intermediate [19]. It can be reasonably deduced that this process could be effectively suppressed by replacing the N–glycosidic linkage with a more stable C–C linkage. This could be achieved by employing a C-nucleoside analog of NAR. Nevertheless, it may be a synthetically rather challenging process. In addition, the inductive effect of substituents in 2′-substituted nicotinamide arabinosides (Figure 3; **21**) [20] was analyzed, supporting a dissociative mechanism involving a cationic intermediate and suggesting that electron-withdrawing substituents retard the rate of anomeric displacement by destabilizing the oxocarbenium ion. The other degradation pathway is based on the fact that the pyridinium ring is susceptible to degradation by the attack of various C-, N-, O-, and S-nucleophiles (for example, cyanide, alkoxide/hydroxide ions, hydroxylamines, sulfides, etc.) on carbon 2/6 and 4 [21]. It has been reported that polarizable ions, such as cyanide, iodide, sulfide, and dithionite preferentially give 1,4-addition products, whereas less polarizable ions, such as hydroxide, alkoxide, or hydride give 1,2- and 1,6-addition products [22]. This is consistent with the observed regioselectivity of dithionite-induced reduction, which mainly yields 1,4-dihydropyridine, whereas borohydride preferentially yields 1,6- and 1,2-dihydropyridine [23]. Subsequent reactions may follow the nucleophilic attack, which typically involves the opening and re-closure of the ring, to generate various degradation products [24,25]. However, this is not a cause for concern in general, given that these reactions are reversible.

It is noteworthy that under strong alkaline conditions (above pH 12.5), the nucleophilic attack of hydroxide ions on the pyridinium ring dominates over the above-mentioned glycosidic hydrolysis. The process is initiated by a sophisticated mechanism, which ultimately results in the formation of 2-pyridone-3-carbaldehyde as the primary product. This may subsequently degrade into various species [19,26]. It is noteworthy that stable adducts of NAR have also been described, which can be reconverted under appropriate conditions. Consequently, stable NAR adducts may prove a valuable alternative to the challenging cationic NAR or NARH in chemical synthesis strategies. For instance, the cyanide adduct of NAR monophosphate (NAR–CN) was employed for the enzymatic synthesis of NAR–CN-containing oligonucleotides, which were subsequently transformed into poly-NAR oligonucleotides by incubation in aqueous buffer. In contrast, the utilization of the parent nucleotide, NARH monophosphate, yielded markedly inferior results. Nevertheless, from a modern perspective, the analytical characterization appears to be insufficient and at times less convincing [27].

### 2.2. Dihydronicotinamide Riboside (NARH) Is Prone to Degradation by Acid-Catalyzed Addition and Nucleophilic Displacement of the Dihydropyridine Moiety in Alkaline Media

**Figure 4:**
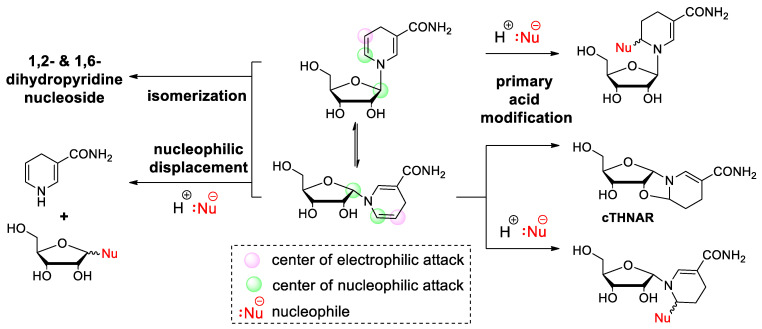
Acid-catalyzed vinylamine addition, anomeric displacement, and isomerization leading to degradation of NARH.

As anticipated, 1,4-dihydronicotinamide riboside is readily oxidized by a plethora of oxidants, including iodine, oxygen, selenium dioxide, permanganate, and various nitrates, amongst others [28,29,30,31,32]. In contrast to the well-known fact that NARH will slowly re-oxidize to NAR if exposed to humid air, its tendency for isomerization to 1,2- and 1,6-dihydronicotinamide riboside under these conditions is rather unknown. Nevertheless, following an extended period of incubation, an initially pure sample of NARH may exhibit a minimal presence of these isomers, up to 4%. These differ widely in stability compared to the parent compound and are potent inhibitors for NAD(P)H-dependent oxidoreductases, indicating their antagonistic role in maintaining cellular redox homeostasis [23]. Both the 1,2-isomer and the 1,6-isomer decompose rapidly, as previously noted [29]. Similarly, NARH is also susceptible to dissociative degradation under alkaline conditions, as previously discussed in the context of NAR. However, the latter is undoubtedly more susceptible to hydrolysis in terms of the leaving group quality. Nevertheless, NARH also undergoes significant degradation under aqueous-alkaline conditions, forming dihydronicotinamide and D-ribose by nucleophilic attack of hydroxide on the anomeric carbon [33] (Figure 4). It is essential to consider this side reaction when adjusting the pH in an experiment. It is notable that this presents a challenge for the chemical reduction of NAR with dithionite, a standard procedure for the synthesis of 1,4-NARH. Even a slight increase in pH (from 8.1 to 8.5) has been observed to increase dihydronicotinamide dissociation, a finding that is evident from a mechanistic perspective. During the course of the reaction, the sulfinate intermediate is relatively stable under alkaline conditions. However, it eventually requires protonation in order to form NARH. Consequently, an increase in pH hinders the nucleophilic replacement at the anomeric carbon by simultaneously formed anions (i.e., thiosulfate, bisulfite), which are more nucleophilic than hydroxide. Consequently, the reduction with dithionite indirectly promotes the hydrolysis of the glycosidic bond, prolongs the reaction time and leads to the accumulation of the 1,6-isomer [33]. In particular, the vinylamine structure of the dihydropyridine ring defines its stability and reactivity. Consequently, the enamine carbon atom is susceptible to electrophilic attack, whereas the carbonyl-analogous position undergoes nucleophilic addition [34]. The presence of electron-donating substituents attached to the enamine nitrogen or enamine carbon, respectively, has been demonstrated to facilitate this reaction [35]. It is well established that 1,4-dihydropyridines degrade under aqueous conditions by hydration of the 2,3-double bond. In contrast, the 5,6-double bond appears to be either unaffected or less reactive. This may be due to the presence of the electron-withdrawing carbamoyl group, which disfavors the initial protonation of the 3 position. NARH, as the representative structural moiety of the genuine co-factors NAD(P)H, is rapidly hydrated to form 2-hydroxy-1,4,5,6-tetrahydronicotinamide riboside (2OH-THNAR), in acidic and neutral media (Figure 4). The acid-catalyzed nucleophilic addition is typically accompanied by a characteristic hypochromic shift of the absorption maximum, from 340 nm to 290 nm [23,36]. This ultimately restricts the range of viable reaction conditions, with implications for biochemical applications, particularly in the context of buffer composition. In practice, however, this is not a significant concern because under physiological conditions, this side reaction is relatively slow and negligible during measurements. Indeed, stability studies have demonstrated that complete degradation occurs immediately at low pH, but is tolerable under physiological or alkaline conditions. Moreover, the integrity of NARH remains intact even after prolonged incubation [33]. Although previously known, the 2′-hydroxyl group of the sugar moiety has been overlooked in recent years. This group is also capable of undergoing acid-catalyzed enamine addition, which leads to the formation of cTHNAR. Due to its ribo configuration, this process is only possible following ring opening, passing through an imine intermediate and subsequent formation of the alpha anomer. The cyclisation process may compete with the hydration of the beta anomer (formation of 2OH-THNAR), depending on the buffer substance, concentration, and pH. At low pH (pH < 3), anomerization occurs at a faster rate than hydration, resulting in the irreversible formation of cTHNAR [37]. In contrast, in neutral or alkaline media, hydration is the predominant process. It is noteworthy that hydration occurs at comparable rates in the majority of solvents and buffer systems, with the exception of the presence of several ions, such as acetate [33] or phosphate, where hydration/addition of the 2′-hydroxyl group is significantly faster [38]. Although the synergistic buffer effect of ions on the hydration of NARH is well established, the underlying mechanism has not yet been definitively elucidated. Furthermore, the formation of additional degradation products under acidic conditions has been described in the literature [39,40]. Nevertheless, these equilibrium reactions in buffers and solvents appear to be insignificant in comparison to the primary acid modification (i.e., the formation of 2OH–THNAR and cTHNAR) (Figure 4).

**Figure 5:**
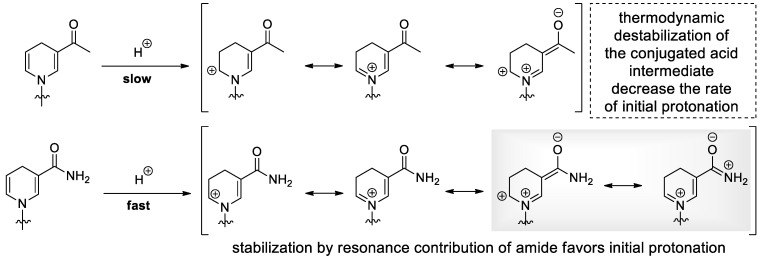
Alteration in resonance contribution of the carbonyl substituent of NARH affecting the rate of initial protonation. The stabilization induced by an additional resonance structure facilitates protonation of the nicotinamide moiety.

Over the years, various attempts have been made to overcome this problem through the synthesis of derivatives and mimetics. For example, the hydration of the dihydropyridine ring can be effectively reduced by substituents. Electron-donating substituents generally increase acid lability, while electron-withdrawing groups suppress acid-catalyzed degradation, but may result in base labile compounds. Substituents on ring carbons or annulated aromatic rings that act as a permanent protective group of the 2,3-double bond are usually accompanied by drastic changes in the redox potential and may lead to loss of function, as observed in enzymatic studies [41,42]. Furthermore, the modification of the 5-substituent, which affects the reactivity of the dihydronicotinamide moiety through mesomeric effects, will have an impact on the overall stability (Figure 5). Consequently, N-benzyl dihydronicotinamide derivatives with variations of the carbonyl group were synthesized and the rate of the primary reaction with acid was determined. In comparison to nicotinamide, any substituent that decreases the thermodynamic stability of the conjugated acid intermediate retards the rate of acid-catalyzed degradation and thus increases the stability against hydration [43].

It was also shown that, similar to the adenosyl residue in NAD(P)H, an aromatic substituent attached to the ring nitrogen can improve stability through π–π-stacking interactions. It has been reported that biomimetic co-factors of NAD containing a phenyl ring with alkyl chains of varying lengths exhibit differences in pH and temperature stability. The mimetic containing an ethyl chain demonstrated stability comparable to NADH at elevated temperatures and low pH. In contrast, derivatives with shorter or longer alkyl chains were less stable due to unfavorable molecular geometry or non-ideal stacking distance [44]. Recently, the carbocyclic derivative carbo NADH, which has a cyclopentane ring instead of ribofuranose, was shown to be almost unaffected by hydration under acidic, neutral, and alkaline conditions (pH 3–10.5). It also has increased thermal stability compared to NADH and is not susceptible to buffer-assisted hydration [45]. The increased stability against nucleophilic substitution of the heterocycle is intuitively understandable for both the oxidized and reduced forms, since, unlike the glycosidic acetal bond, the dissociation of the heterocycle is not supported by neighboring group effects. Nevertheless, the improved hydration stability of the reduced species is truly remarkable. However, the underlying structure–reactivity relationship that gives rise to this extraordinary stability is still under investigation.

## 3. Synthesis of Nicotinamide Riboside

### 3.1. Synthetic Paths to Nicotinamide Riboside

**Figure 6:**
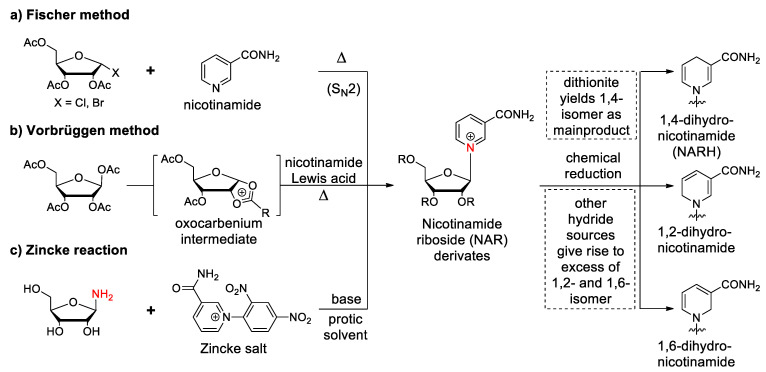
Access to NAR(H) derivatives by (**a**) nucleophilic substitution of halo sugars; (**b**) Lewis acid catalyzed glycosylation of per-acetylated ribose, and (**c**) formation of the nicotinamide moiety through Zincke reaction.

Due to the stability issues of NAR discussed above, incorporation of the reduced form, NARH, into RNA followed by oxidation may be a possible way to provide the redox co-factor within and covalently bound to an RNA structure. To do this, NARH needs to be synthesized, followed by decoration with the typical functionalities, to convert it into a phosphoramidite building block. One way to synthesize the nucleoside and its derivatives is to glycosylate nicotinamide with a suitable ribose derivative. Early methods include the use of *O*-acyl-protected halosugars (Figure 6a). These mostly tri-*O*-benzoyl or -acetyl halo ribofuranosides can then be condensed with nicotinamide to give the corresponding tri-acyl nicotinamide nucleoside, which can be deprotected using methanolic ammonia, whether the reduced or oxidized species [46,47]. The selectivity of glycosylation tends to yield the desired β-anomer with approximately 90% yield, while yields of up to 5% of the α-anomer have been reported [48,49]. The reason for the stereoselectivity has been attributed to the formation of an intermediate 1,2-acyloxonium sugar, which is formed by anchimeric assistance of the 2-*O*-acyl group [50]. Without the acyl groups, the resulting nucleoside would be racemic.

Another strategy is to use a Lewis acid such as trimethylsilyltrifluoromethane sulfonate (TMSOTf) to catalyze glycosylation (Figure 6b). Here, TMSOTf reacts with the per-acetylated ribose to form the 1,2-acyloxonium sugar mentioned above. This facilitates the condensation of the sugar with nicotinamide to give tri-acetyl nicotinamide riboside. As with the previous strategy, the β-anomer is the preferred product, but it is reported that approximately 13% of the α-anomer is formed [51]. In order to suppress the appearance of this unwanted by-product, an optimized version of this reaction has been developed. The key element of this procedure is the silylation of nicotinamide with trimethylsilyl chloride and the use of catalytic amounts of TMSOTf in the subsequent coupling with per-acetylated ribose. Under these controlled conditions, the beta-anomer appears as the sole product [52].

Instead of using nicotinamide, ethyl or methyl nicotinate can be condensed with tetra-acetyl ribose in the presence of TMSOTf. The resulting cationic nicotinate ester can then be treated with methanolic ammonia, resulting in simultaneous de-acetylation of the hydroxy groups and ester to amide conversion to give beta-nicotinamide riboside [53,54].

The Zincke reaction is another possibility for the chemical synthesis of nicotinamide riboside. Nicotinamide, a pyridine derivative, can react with an electron-poor aromatic compound such as 2,4-dinitrochlorobenzene to form the Zincke salt (Figure 6c). This salt can then be condensed with 1-amino sugars to form the corresponding nicotinamide nucleoside. It is noteworthy that the absolute stereo-configuration of the anomeric carbon remains the same, since the nucleobase is generated from the sugar moiety which has a defined stereo-configuration [55].

Reduction of the pyridinium moiety can be carried out with or without protection of the hydroxyl groups. However, if the reaction is carried out in an aqueous system, NARH can be extracted with an organic solvent as long as the hydroxyl groups are acylated. This greatly facilitates purification after reduction. The general procedure is to dissolve tri-acetylated nicotinamide riboside in dichloromethane and mix it with an aqueous solution of NaHCO_3_ and Na_2_S_2_O_4_. After a few hours the organic layer containing the reduced nucleoside can be separated. After removing the acetyl groups with methanol and K_2_CO_3_, NARH is obtained [56]. As mentioned above the choice of the reducing agent is crucial for this reaction, as three different isomers of NARH can be formed under different conditions. The only stable and biologically relevant isomer, 1,4-dihydronicotinamide, is the main product when sodium dithionite is used as the reducing agent [23]. This regioselectivity is due to the particular orientation of the dithionite anion to the pyridinium salt, which determines the position of attack as carbon 4. It is supported by the distance between the two nucleophilic oxygen atoms of dithionite, which is very similar to the interatomic distance between the ring nitrogen and the electrophilic C4 atom [57]. Interestingly, when performing the dithionite-mediated reduction with NAR mimetics, this regioselectivity disappears [58]. Moreover, if a stronger reducing agent such as NaBH_4_ is used, a mixture of the 1,2-, 1,4-, and 1,6-isomers is obtained in a ratio of 1:0.44:1 [56]. The hydride anion has the potential to attack any of the three carbon atoms in the pyridinium ring. However, selectivity is observed towards the 1,2- and 1,6-isomers due to their weak polarizability, making them more favorable targets for the hydride anion compared to carbon 4.

### 3.2. Synthetic Modification of the Structure of the Ribose Moiety

As discussed in Section 2, the limited stability of nicotinamide riboside is a consequence of its readily cleavable glycosidic bond. This must be taken into account when attempting to convert the nucleoside derivative into a phosphoramidite building block for incorporation into RNA by solid phase synthesis. Functional group protection and phosphoramidite synthesis involve multiple chemical reaction steps that are prone to degradation of NAR. To circumvent this problem, several NAD mimetics with different properties have been developed.

**Figure 7:**
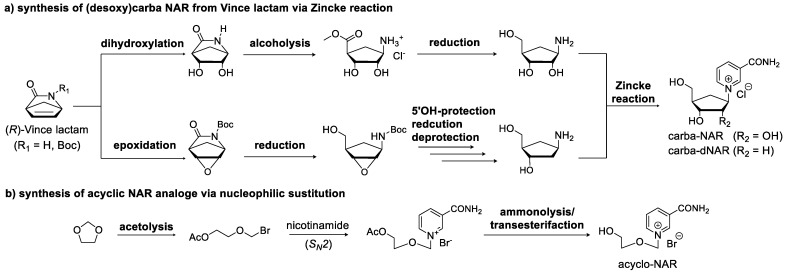
Synthetic strategies towards modified sugar moieties. (**a**) Preparation of carbocyclic nicotinamide nucleosides. (**b**) Prototypic synthesis of an acyclic NAR analog.

It has already been shown that the alternative nucleoside-structured carbocyclic NAR has greatly increased stability compared to natural NAR at various pH values. Therefore, carbocyclic nicotinamide (Figure 3; **19**) [59] can be used to maintain the general idea of incorporating nicotinamide as a nucleoside into RNA via solid phase synthesis. The carbocycles of the canonical bases have previously been shown to be functionalized as phosphoramidites and incorporated into RNA via solid phase synthesis [60]. Carbocyclic sugar analogs (Figure 3; **16**, **17**) [61,62] are synthesized from (*R*)-Vince lactam, which is first converted to amino carba–ribose. It can then react with the corresponding nicotinamide Zincke salt to form nicotinamide carba-riboside (Figure 7a) [59].

The same lactam can also be used to prepare carbocyclic deoxyribonucleosides [61]. The reaction pathway slightly differs from that of the ribonucleosides, resulting in a different precursor. However, the final step also involves the aforementioned Zincke reaction. Recently, the chemoenzymatic replacement of the endocyclic ribose oxygen with a less electronegative sulfur has been achieved, resulting in nicotinamide 4′-thioriboside (Figure 3; **18**) [63], with reduced reactivity of the N-glycosidic linkage while maintaining the riboside geometry.

Another modification that can be made to the ribose structure is to change from the typical ring sugar to an acyclic sugar, accessible by simple nucleophilic substitution from the corresponding halide (Figure 3; **1**–**8**) [64,65,66,67,68,69,70,71]. The absence of the 2′- and 3′-hydroxy groups facilitates the functionalization of the nucleoside for use in chemical RNA synthesis. For example, a prototypical synthesis of an acyclic nucleoside involves the reaction of a linear halide with nicotinamide. The acyclic sugar analog can be synthesized by acetolysis of 1,3-dioxolane with acetyl bromide. The resulting alkyl halide can then be reacted with nicotinamide to give acetylated pyridinium bromide. Subsequent deacetylation in methanolic ammonia yields the acyclic nicotinamide riboside (Figure 7b; Figure 3; **4**) [67,72].

### 3.3. Synthetic Modifications of the Nicotinamide Base

**Figure 8:**
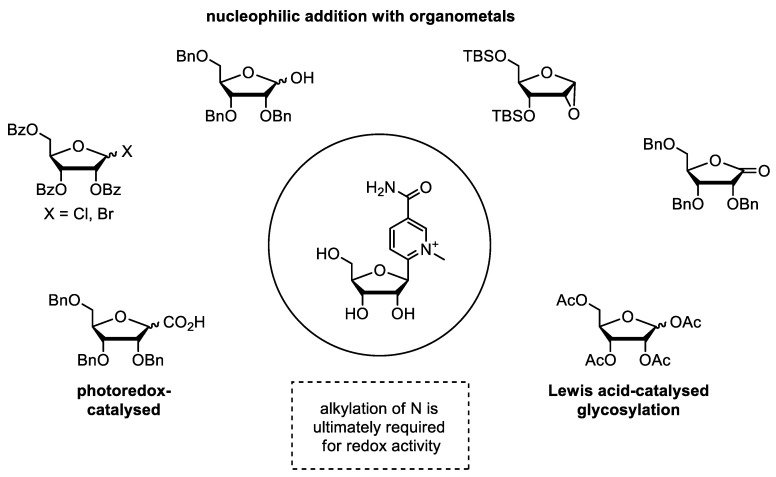
General strategies for the synthesis of nicotinamide derived C-ribonucleosides. After formation of the nucleosidic bond, an alkylation of the ring nitrogen is required for redox activity.

In addition to structural modifications of the ribose sugar, the glycosidic linkage can also be strengthened by altering the nicotinamide moiety. One promising option is to replace the N-glycosidic bond with a C-glycosidic bond, which significantly increases its stability. There are a number of ways to make such a carbon link, and pyridine C-nucleosides have already been synthesized. One way is to use a fully protected ribonolactone which reacts with lithiopyridine to give the corresponding hemiacetal (Figure 8). This intermediate can then be reduced to the corresponding C-ribonucleoside [73].

Other methods include Friedel–Crafts glycosylation with aromatic compounds and acetylfuranoses, photoredox coupling of furanosyl acids with aryl bromides, and nucleophilic additions [74,75,76,77,78]. For the synthesis of the nicotinamide C-nucleoside, it is important to choose a pyridine derivative with a functional group in position 3 that can be converted to a carbamoyl group, such as bromine or nitrile [73,79,80]. In addition, the ring nitrogen of the C-nucleoside must be alkylated, for example, with methyl iodide, to exhibit redox activity similar to that of N-glycosidic NAR [79].

## 4. Attempts to Incorporate Nicotinamide Riboside in RNA

### 4.1. Synthesis of NAR/NARH Building Blocks

**Figure 9:**
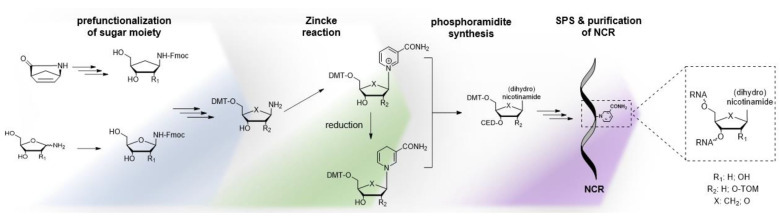
Proposed strategy for synthesis of NCRs by the phosphoramidite approach. The Zincke reaction enables access to partially protected nucleosides from pre-functionalized sugar moieties.

**Figure 10:**
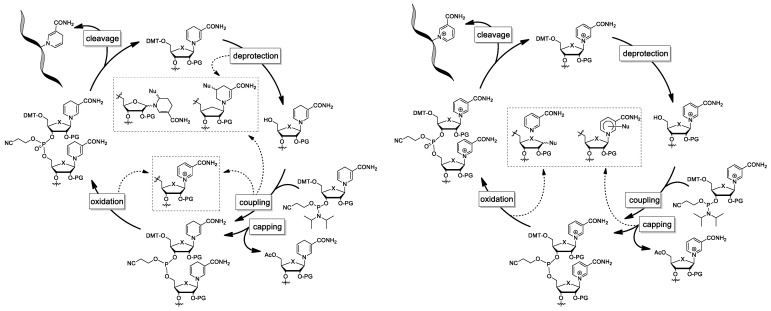
Hypothetic outcome of chemical solid phase synthesis using NARH (**left**) or NAR (**right**) phosphoramidite. Dashed arrows point to possible degradation products; X: O, CH_2_.

Obviously, the site-specific incorporation of nicotinamide riboside into RNA is by no means trivial. To date, attachment to the 5′- and 3′-terminus has been reported, but solid-phase synthesis (SPS) approaches for the synthesis of nicotinamide-containing oligoribonucleotides (NCRs) have not yet been demonstrated. Efforts to modify the 5′-terminus have been driven primarily by studies of the so-called 5′-NAD cap. These studies provide access to ligation of nicotinamide mononucleotide (NMN) to the 5′-terminus of oligonucleotides via a pyrophosphate linkage instead of the natural phosphodiester bond, using a large excess of activated intermediates such as NMN-imidazolide (Figure 3; **11**, **12**) [81,82]. Among these NMN amidates, conjugation via the activated nicotinamide carbamoyl group is feasible (Figure 3; **9**, **10**) [83] and has been used several times as a straightforward approach for the construction of quite intriguing redox relays [84]. In addition, several azido- or alkyne-modified nicotinamide derivatives (Figure 3; **13**, **14**, **15**) [85,86,87] are described, suggesting conjugation via alkyne–azide cycloaddition or Staudinger ligation, respectively. However, both approaches, active ester and click chemistry, are of little avail for NCR synthesis, when structural similarity to canonical incorporated nucleotides is demanded. Transcriptional priming with excess NAD or tethering of NMN by an in vitro selected variant of the hairpin ribozyme catalyzing the addition of NAD-2′,3′-cyclic phosphate has also been described [88]. Göckel and Richert reported primer extension with NMN-imidazolide, which is attached to the 3′-terminus via a phosphoramidate linkage. This linkage is isoelectronic to the natural phosphodiester bond, although it exhibits slightly altered stereo-electronic properties [82,89]. Decades ago, the enzymatic de novo polymerization of NMN and primer extension using the di- and triphosphates of reduced NMN (NARH-5′-monophosphate) and NMN–CN were reported to be catalyzed by polynucleotide phosphorylase [27]. While the reduced species and the cyanide adduct were found to be suitable substrates, the oxidized form was less efficient, but instead was successfully used to extend a DNA primer by one or two nucleotides in the presence of terminal deoxyribonucleotide transferase [27].

Although it has been claimed that the lability of NAR and NARH towards bases or acids precludes the SPS of NCRs, there is as yet no convincing experimental evidence to support this claim. Instead, we argue the opposite, as preliminary results from our laboratory suggest that the synthesis of NCRs using the phosphoramidite approach could be feasible through sophisticated processing. This is further supported by the fact that key compounds such as 5′-*O*-trityl protected NAR (Figure 3; **22**) [90] and NARH phosphoramidite (Figure 3; **23**) [91] have been described previously. Nevertheless, we would like to point out that the attempts to incorporate NAR or NARH into oligonucleotides present several synthetic challenges. Based on our own observations, we can confirm that the synthesis of phosphoramidite building blocks starting from commercially available NAR or NARH is associated with serious problems, as the conditions required for the incorporation of protective groups lead to side reactions and decomposition of NAR or NARH due to their inherent instability. The key to the synthesis of such building blocks is the formation of the glycosidic bond by the Zincke reaction using an amino sugar with the required 5′-*O*-trityl and 2′-*O*-protective groups [90]. For this approach, the use of a base-stable 2′-*O*-protecting group, such as the triisopropylysilyloxymethyl (TOM) group, rather than the commonly used *tert*-butyl dimethylsilyl (tBDMS) group, is mandatory, as silyl group migration is certainly inevitable under these strong basic conditions. However, in view of the above-mentioned degradation pathways of NARH, it would be reasonable to carry out such a synthesis with a 2′-deoxyamino sugar or the corresponding carbon cycle (Figure 3; **16**, **20**) [20,61], starting from the Vince lactam (Figure 9) [62]. Finally, the phosphoramidite group (i.e., the CED group) is introduced. This approach should also be applicable to the synthesis of building blocks for the H-phosphonate or phosphotriester method.

### 4.2. Application of a NAR/NARH Phosphoramidite

Hypothetically, when using a NAR phosphoramidite in oligonucleotide synthesis, we predict that the heterocycle will be unaffected during detritylation and coupling, as it is stable under acidic conditions. In contrast, reagents that require basic processing (i.e., capping and oxidation) could lead to degradation. However, stability studies have shown that even after prolonged incubation of NAR with the oxidation reagent (i.e., iodine), no reaction or degradation was observed in the solvents used (ACN, trimethylpyridine, water) (unpublished data). Decomposition as a result of capping is not to be expected either, since although the process is carried out under basic conditions, only poor nucleophiles are present (Figure 10). Therefore, the use of a NAR phosphoramidite seems to be feasible. However, an effect on the solubility of the phosphoramidite is to be expected due to the cationic nature of the nicotinamide moiety. This may be an issue during SPS, but should be easily overcome by the non-polar-protecting groups present in a fully functionalized phosphoramidite. Nevertheless, the extent to which the cationic pyridinium moiety influences subsequent coupling after its incorporation and partial deprotection is still unexplored. However, it is known that the nucleophilicity of the 5′-hydroxyl group is reduced compared to canonical nucleosides due to electrostatic interactions with the pyridinium moiety [92]. This would adversely affect the coupling efficiency of the subsequent building blocks, but could possibly be compensated by prolonged coupling times and/or multiple coupling cycles. Nevertheless, the fact that 5′-silylation of NAR with sterically hindered tri-isopropylsilyl chloride (Figure 3; **24**) and subsequent deprotection with TBAF was achieved suggests a promising outcome during the coupling step and moreover indicates the compatibility of NAR with silicon-based protection group chemistry and conventional deprotection with fluoride reagents [93].

When using NARH phosphoramidites, it is expected that the dihydropyridine moiety will be immediately reconverted to the pyridinium species (i.e., NAR) during the oxidation step. Interestingly, we observed that NARH is indeed oxidized in quantitative yield, but no further degradation of the reduced or oxidized species was observed (unpublished data). Again, the oxidation of the dihydropyridine moiety is likely to be negligible as long as the coupling of the next building block is not affected. Alternatively, chemoselective oxidation of the phosphite could be carried out with hydrogen peroxide in aqueous methanolic solution, leaving the dihydropyridine moiety virtually intact [91]. Interestingly, oxidation of the dihydropyridine moiety was subsequently mediated by cobalt acetate under the same conditions [91]. Exploratory experiments also showed that NARH is inert towards the capping reagent. A more serious issue is the expected degradation of the dihydropyridine moiety during acidic processing (i.e., detritylation and coupling/activation). As expected, we observed the formation of cTHNAR from NARH upon exposure to the detritylation reagent (dichloroacetic acid in dichloroethane) (unpublished data). Hypothetically, this cyclization is blocked in a NARH phosphoramidite by the 2′-*O*-protecting group. Nevertheless, anomerization and formation of tetrahydropyridine derivatives by addition of nucleophiles is likely to occur. However, de-tritylation using Lewis acids such as zinc halides [94], offers a simple way to overcome this hurdle, as no degradation was observed after prolonged incubation of NARH in zinc chloride/dichloroethane (unpublished data). As the activation step is also acidic, degradation of the dihydropyridine might also be expected. However, this was not observed. To our surprise, incubation of NARH with benzylmercaptotetrazole (BMT), currently the most commonly used activator for phosphoramidite coupling, leads to oxidation (i.e., formation of NAR) (unpublished data). In principle, this is in line with widely used colorimetric assays for the characterization of NAD(P)H-dependent dehydrogenases by the formation of formazan derivatives from tetrazolium salts (e.g., MTT assay) [95]. Surprisingly, tetrazole has been successfully used for phosphoramidite coupling with 2′,3′-protected NARH, suggesting the use of alternative activators, although tetrazole is certainly a less efficient variant [91].

After successful synthesis, the oligonucleotide must be completely deprotected and cleaved from the solid support to allow further purification. Normally, this is performed under highly basic conditions with an ammonia/methylamine solution at elevated temperatures to ensure complete removal of the highly stable nucleobase-protecting groups. For NCRs, however, these conditions would need to be adjusted to prevent degradation of the sensitive nicotinamide moiety. As mentioned above, deprotection of an acetylated nicotinate ester was achieved by exposure to 5.5 N methanolic ammonia solution at 0 °C for 15 h, allowing NAR to be isolated in high yield [54]. Therefore, deprotection under mild basic conditions would be possible. In addition, the use of less stable amino-protecting groups and solid-phase linkers, i.e., acetyl or iso-butyryl groups and an oxalyl linkage, may help [96]. Using 50 mM K_2_CO_3_ in anhydrous methanol, it is also possible to rapidly cleave the labile-protecting groups from oligonucleotides containing fragile modifications, thereby delaying the degradation of such modifications [97].

Another option is to implement orthogonal strategies such as fluoride–labile-protecting groups. These groups can be rapidly removed by treatment with various fluoride reagents, as has been demonstrated for nucleobase- and phosphate-protecting groups [98]. Since de-silylation with TBAF was successfully performed for NAR at the nucleoside level, we argue that this is a convenient and straightforward method even when using a NARH phosphoramidite, since the reduced form is less susceptible to alkaline degradation than NAR. Considering that the oxidized form of NCR is the expected outcome after SPS as a result of the oxidation step, this approach seems to be applicable regardless of whether NAR or NARH phosphoramidite is used.

## 5. Conclusions

Cofactor-dependent ribozyme activity is a compelling scenario that has been widely described in the literature, including redox-active co-factors such as FMN or NAD [99,100,101]. For the further development of redox-active ribozymes, the incorporation of co-factors mediating electron transfer processes into RNA is a highly desirable goal, as it would pave the way for ribozyme-catalyzed enantioselective and regioselective redox reactions and provide the basis for novel biotechnological platforms. Much effort has been devoted to the incorporation of NAR or NARH into RNA. This is still a challenging task, due to the limited stability of the oxidized species (NAR) in the presence of bases and the reduced species (NARH) in the presence of acids. Another serious problem is the susceptibility of C1′ and the pyridinium moiety to attack by a nucleophile, leading to decomposition. Similarly, NARH is susceptible to degradation by acid-catalyzed nucleophile addition. Although in principle possible through glycosylation reactions, the synthesis of monomeric NAR(H) is a challenging endeavor, due to the stability problems. The use of carba–ribose and/or C-linked nucleosides may be a way to circumvent the stability problems, and a promising avenue for future investigations. It remains to be shown whether NAR(H) can be transferred to a phosphoramidite building block for use in solid-phase oligoribonucleotide synthesis. It seems possible if the reaction conditions are carefully controlled and adjusted. Key intermediates such as NARH-5′-phosphoramidite or 5′-*O*-DMT protected NAR have already been synthesized [90,91], and the key steps of oligonucleotide synthesis have been successfully performed with similar compounds [54,93,96,97,98]. Therefore, we are confident that the incorporation of NAR(H) into oligonucleotides will be successful in the near future. 

## Data Availability

Not applicable.
